# Bone marrow microRNA-34a is a good indicator for response to treatment in acute myeloid leukemia

**DOI:** 10.32604/or.2023.043026

**Published:** 2024-02-06

**Authors:** MONA S. ABDELLATEIF, NAGLAA M. HASSAN, MAHMOUD M. KAMEL, YOMNA M. EL-MELIGUI

**Affiliations:** 1 Department of Cancer Biology, Medical Biochemistry and Molecular Biology, National Cancer Institute, Cairo University, Cairo, 11976, Egypt; 2 Department of Clinical Pathology, National Cancer Institute, Cairo University, Cairo, 11976, Egypt

**Keywords:** AML, miR-34a, microRNA, Leukemia

## Abstract

**Background:**

microRNA-34a (miR-34a) had been reported to have a diagnostic role in acute myeloid leukemia (AML). However, its value in the bone marrow (BM) of AML patients, in addition to its role in response to therapy is still unclear. The current study was designed to assess the diagnostic, prognostic, and predictive significance of miR-34a in the BM of AML patients.

**Methods:**

The miR-34a was assessed in BM aspirate of 82 AML patients in relation to 12 normal control subjects using qRT-PCR. The data were assessed for correlation with the relevant clinical criteria, response to therapy, disease-free survival (DFS), and overall survival (OS) rates.

**Results:**

miR-34a was significantly downregulated in AML patients [0.005 (3.3 × 10^−6^–1.32)], compared to the control subjects [0.108 (3.2 × 10^−4^–1.64), ***p* = 0.021**]. The median relative quantification (RQ) of miR-34a was 0.106 (range; 0–32.12). The specificity, sensitivity, and area under the curve (AUC) for the diagnosis of AML were (**58.3%**, **69.5%**, **0.707**, respectively, ***p* = 0.021**). patients with upregulated miR-34a showed decreased platelets count <34.5 × 10^9^/L, and achieved early complete remission **(**CR, ***p* = 0.031**, ***p* = 0.044**, respectively**)**. Similarly, patients who were refractory to therapy showed decreased miR-34a levels in comparison to those who achieved CR [0.002 (0–0.01) and 0.12 (0–32.12), respectively, ***p* = 0.002**]. Therefore, miR-34a could significantly identify patients with CR with a specificity of 75% and sensitivity of 100% at a cut-off of 0.014 (AUC = 0.927, ***p* = 0.005).** There was no considerable association between miR-34a expression and survival rates of the included AML patients.

**Conclusion:**

miR-34a could be a beneficial diagnostic biomarker for AML patients. In addition, it serves as a good indicator for response to therapy, which could possibly identify patients who are refractory to treatment with 100% sensitivity and 75% specificity.

## Introduction

Acute myeloid leukemia (AML) is the commonest leukemia that affects both adults and pediatrics. It is a haematological neoplasm that is characterized by uncontrolled proliferation and differentiation of hematopoietic precursor cells, which results in increased number of immature myeloblasts in the bone marrow (BM) and the peripheral blood (BP) of the patients [1,2]. In 2021, the American Cancer Society reported that AML was the second most commonly diagnosed leukemia and it was the leading cause of leukemia-related death in the United States [3]. Though the advancement in the treatment strategies of AML, still there is an increased frequency of relapse and short overall survival (OS) rates, especially in older ages [4,5]. Therefore, it is highly required to search for other biological markers that can be used for early detection, diagnosis, and prognosis of AML patients.

The pathogenesis of AML is a multistep process, which included different genetic, epigenetic, and environmental factors [6]. The most commonly found genetic mutations that contributed to the development of AML are tumor protein 53 (*TP53*), internal tandem duplication of FMS-like tyrosine kinase 3 (*FLT3-ITD*), nucleophosmin 1 (*NPM1*), and *CCAAT*/enhancer-binding protein alpha (*CEBPA*) [7]. Thus, the recent risk classifications of the AML including the world health organization (WHO) and European LeukemiaNet (ELN) stratification rely mainly upon the associated molecular aberrations in combination with the related cytogenetic abnormalities [7–9].

microRNAs (miRNAs) are small, non-coding RNAs that comprise 22–25 nucleotides. it negatively regulates gene expression by different mechanisms including binding to the 3′-untranslated regions of mRNA or interfering with its translation [10]. There are many miRNAs such as miR-55, miR-210, miR-96, and miR-328, which had been identified to have essential functions in the initiation and promotion of AML [11–14]. Additionally, the miR-199b had a prognostic role in AML patients [15]. Another miRNA aberration that had been detected in AML development was the miR-34a, which belongs to the miR-34 family, and it is found at chromosome 1p-36.23 [16]. It is the first identified miRNA that had a tumor suppressor function, and it was found to be downregulated in many tumors including colorectal, breast, as well as lung cancer [17–19]. It had been reported by many recent series that miR-34a has a crucial role in p53- stimulated cell cycle arrest, apoptosis, and other pathways regulating the cellular biological process [20,21].

It had been reported that there was an increased incidence of disease relapse in AML patients especially those with old age. Therefore, it is essential to search for a biological marker that could predict patients’ response to treatment. Hence, it will allow for shifting to another therapeutic modality in those unresponsive cases, and consequently, protect them from unneeded side effects. The current study aimed at evaluating the biological importance of miR-34a in the diagnosis, prognosis, and outcomes of AML patients. This was performed by assessing the association between the miR-34a expression levels in the BM of AML patients and the clinico-pathological characteristics, with regards to the response to therapy, outcomes, the overall survival (OS) as well as the disease-free survival (DFS) rates of the patients.

## Materials and Methods

### The source of the patients

This retrospective cohort study comprised 82 *de novo* AML patients compared to 12 age and sex-coordinated normal healthy individuals. The included patients were presented and diagnosed at the Medical Oncology Department of the National Cancer Institute (NCI), from the period between January 2015 to September 2016. The control samples were obtained from subjects who donated for bone marrow transplantation (BMT) at the NCI. Where informed consents were obtained from both groups before engagement in the study and the study was approved by the Medical Ethical Research Committee of the National Research Centre (No. P100510), which was in accordance with the Helsinki declaration 2011. All patients were subjected to full clinical examination, laboratory, and radiological assessment, in addition to complete history taking. The diagnosis of AML was performed according to the morphological, cytochemical, cytogenetic, and immunophenotyping (IPT) evaluation of the BM aspirate according to the WHO classification of the hematopoietic and lymphoid tissue tumors [8].

### Management of the patients

The therapeutic protocol of the patients included induction therapy which formed of daunorubicin 45 mg/m^2^ and cytosine arabinoside 100 mg/m^2^, followed by two cycles of daunorubicin 45 mg/m^2^ for 2 days, plus cytosine arabinoside 100 mg/m^2^ for another 5 days after remission [22].

The follow-up of the patients consisted of both clinical and BM examinations on day 14 and 28 of the induction treatment. The outcome of the induction treatment was evaluated on day 28, where patients were classified according to their response into patients who were refractory to treatment and patients with complete remission (CR). The CR was reported if the absolute neutrophil count was 1.5 × 10^9^/L, platelet count of 100 × 10^9^/L or more, no blasts in the peripheral blood (PB), BM cellularity more than 20%, no Auer rods, less than 5% BM blasts, and no extramedullary leukemia [7].

### Sample collection and miRNA extraction

A sample of 1 mL BM fluid was aspirated from each patient by iliac or tibial puncture with strict aseptic precautions at presentation before receiving any medication. Samples were collected in a tube containing an anticoagulant and they were managed instantaneously in laminar airflow.

The miRNA was extracted from the BM aspirate of the patients and control subjects using miRNeasy Mini Kit (Qiagen, USA, Cat. no. 217004) as recommended by the manufacturer’s instructions. The extracted miRNA was evaluated for purity and concentration using a spectrophotometer Nano-drop (Maestrogen, Taiwan, MN-913) at 260 and 280 wavelengths, with a ratio of 1.8–2.0 indicated good quality of the miRNA. Gene-specific complementary DNA (cDNA) was formed by using reversing TaqMan microRNA RT-Kit (Applied Biosystems, Foster City, CA, USA, Cat. no. 4366596), according to the manufacturer’s instructions.

### Detection of miRNA expression by real-time PCR

The miRNA expression was quantified using TaqMan 2x universal master mix II (Applied Biosystems, Foster City, CA, USA, Cat. no. 4440043) and TaqMan microRNA Assay Mix containing PCR primers and TaqMan probes for miR-34a. The MiR-u6 20X was used as an endogenous control for the normalization of the samples (Thermofisher ID 001973, Applied Biosystem, USA, California, Cat. no. 4427975). Fluorescence was detected by ABI StepOne Real-Time PCR System (Applied Biosystems Foster City, CA, USA). The amplification reaction was performed using a total volume of 25 µL, with the thermal reactions formed of initial activation for 15 min at 95°C, followed by 40 cycles of denaturation at 94°C for 15 s, annealing at 55°C for 30 s, and extension at 70°C for 30 s. The relative expression of miR-34a was calculated by the comparative Ct method (2^−ΔCt^) [23].

### Statistical methods

The data were analysed by the SPSS© Statistics version 22 (IBM© Corp., Armonk, NY, USA). Normality tests were performed, where the numerical data were presented as median and range, and qualitative variables were presented as frequency and percentage. The association between qualitative variables was done using Fisher’s exact test or Chi-square. Comparison between numerical variables was done using the Mann-Whitney test and Kruskal-Walli’s test. The Area under the receiver operating curve (ROC) was performed to detect the diagnostic power of miR-34a in AML. The disease-free survival was considered from the date of complete remission to the date of relapse, death, or the last follow-up visit. The overall survival (OS) is the period between the date of the diagnosis and the date of death or last follow-up visit. Survival analysis was done by Kaplan-Meier test and log-rank test was used for comparing survival curves. All tests were two-tailed, and the significant level was considered if *p*-value < 0.05.

## Results

The median age of the included AML patients was 37 (range: 18–63) years, and the mean was 37.8 ± 11.8 years. Males represented 47.6% (39/82), and females were 52.4% (43/82). There were 10.9% (9/82) of the patients had diabetes mellitus (DM) and/or arterial blood hypertension. Hypercellular BM was detected in 63 (76.8%) patients, and hypocellular BM was found in 8 (9.8%) patients, while only 11 (13.4%) patients had normocellular BM. Patients were categorized into favourable risk in 16 (19.5%) patients, intermediate risk in 50 (61.0%) patients, and adverse risk in 16 (19.5%) patients, according to the 2017 ELN risk stratification [20]. There were 48 (92.3%) patients who achieved CR, of them, 40 (83.3%) patients showed early CR, and 8 (16.7%) patients had late CR. While there were 4 (7.7%) patients refractory to the treatment, in addition to 8 (10.7%) patients who had relapsed. Finally, there were 47 (57.3%) died, 32 (39%) alive, and 3 were lost for follow-up. The other clinico-pathological characteristics of the patients were shown in Table 1.

**Table 1:** Clinical data of the included AML patients

Variable	Frequency	Variable	Frequency
Age		Gender	
Mean ± SD	37.8 ± 11.8	Male	39 (47.6%)
Median (range)	37 (18–63)	Female	43 (52.4%)
TLC	27 (0.5–440)	PB blast %	50 (0–98)
HB	7.7 (4.3–12.1)	BM blast %	72.5 (14–97)
Platelets	34.5 (2–297)		
BM cellularity		FAB	
Normocellular	11 (13.4%)	M1	14 (17.1)
Hypocellular	8 (9.8%)	M2	32 (39%)
Hypercellular	63 (76.8)	M3 variant	1 (1.2%)
Translocations		M4	23 (28%)
Negative	65 (79.3%)	M5a	6 (7.3%)
Inverted 16	7 (8.5%)	M5b	3 (3.7%)
t (8,21)	8 (9.8%)	M7	3 (3.7%)
t (9,22)	1 (1.2%)		
t (8,21), t (9,22)	1 (1.2%)		
FLT-3		NPM	
Wild	68 (84%)	Wild	17 (68%)
Mutant	13 (16%)	Mutant	8 (32%)
Risk		Organomegaly	
Favorable	16 (19.5%)	No	51 (62.2%)
Intermediate	50 (61.0%)	HM	8 (9.8%)
Adverse	16 (19.5%)	HSM	18 (22%)
		SM	5 (6.1%)
LNs		Response to treatment	
Negative	56 (68.3%)	Refractory	4 (7.7%)
Positive	26 (31.7%)	CR	48 (92.3%)
CR1		CR2	
Negative	10 (20%)	Negative	2 (20%)
Positive	40 (80%)	Positive	8 (80%)
Relapse		Death	
Negative	67 (89.3%)	Live	32 (39%)
Relapse	8 (10.7%)	Died	47 (57.3%)
		Lost follow up	3 (3.7%)

Note: BM: bone marrow, CR: complete remission, FAB: The French-American-British, FLT-3: FMS-like tyrosine kinase 3, HM: hepatomegaly, HB: haemoglobin, HSM: hepatosplenomegaly, IPT: immunophenotyping, LN: lymphadenopathy, NPM: Nucleophosmin, PB: peripheral blood, PLT: platelets, SM: splenomegaly, TLC: total leukocyte count.

### The expression level of miR-34a in AML patients

**Figure 1:**
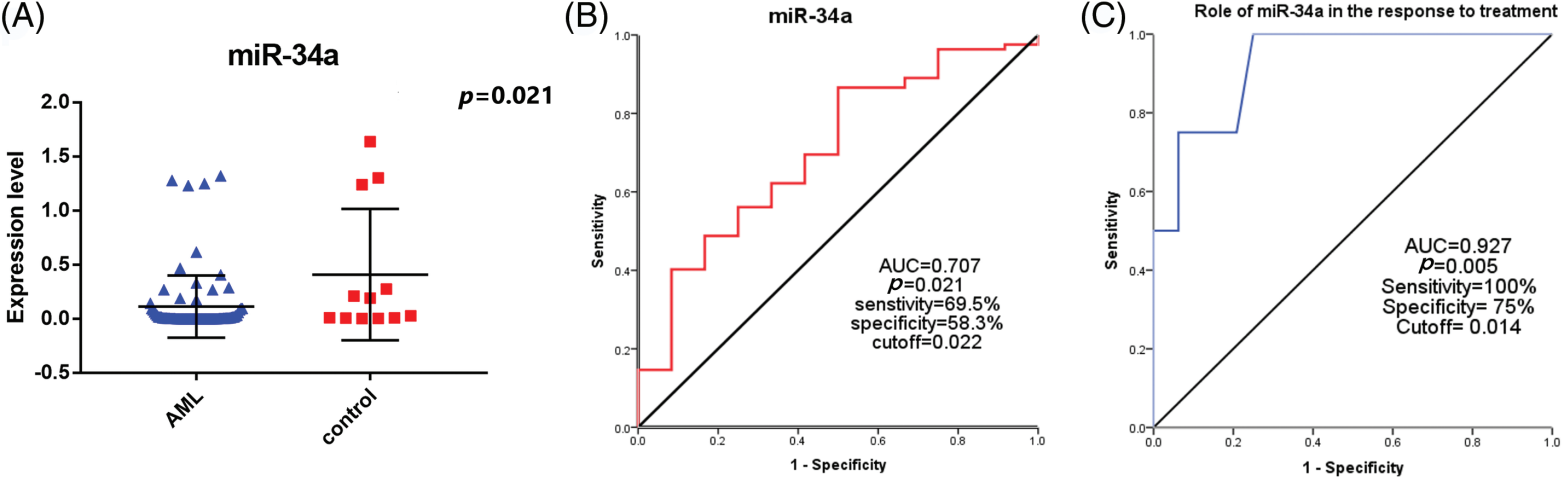
(A) The expression levels of miR-34a in AML patients and the control group, (B) ROC curve analysis of miR-34a expression for the diagnosis of AML patients compared to the control group. (C) Evaluating the role of miR-34a expression in assessing the response to therapy in AML patients using ROC curve analysis.

There was a significant decrease in miR-34a expression in AML patients [0.005 (3.3 × 10^−6^–1.32)], with regards to the control group [0.108 (3.2 × 10^−4^–1.64), ***p* = 0.021**, Fig. 1A]. The median relative quantification (RQ) of miR-34a in AML patients was 0.106 (range; 0–32.12).

### Diagnostic significance of miR-34a in the AML patients

The specificity, sensitivity, and AUC of miR-34a for the diagnosis of AML patients were (**58.3%**, **69.5%**, **0.707**, respectively, ***p* = 0.021**) at a cut-off value of 0.022 according to the performed ROC curve test (Fig. 1B).

### Association between miR-34a expression and the clinical features of the AML patients

There was a significant increase of miR-34a expression in patients with platelet count <34.5 × 10^9^/L in comparison to those with platelet count >34.5 × 10^9^/L [0.18 (0–32.12) compared to 0.03 (0–25.2), respectively, ***p* = 0.031**]. Patients who achieved early CR (before day 28) had increased levels of miR-34a [0.12 (range; 0–32.12)] compared to those who did not achieve early CR [0.02 (range; 0–0.16), ***p* = 0.044**]. Similarly, at the end of the treatment, patients who were refractory to therapy showed decreased levels of miR-34a compared to those who achieved CR [0.002 (0–0.01) and 0.12 (0–32.12), respectively, ***p* = 0.002**, Table 2]. Accordingly, the ROC curve analysis demonstrated that miR-34a could be a useful indicator for CR in AML patients with a specificity of 75% and sensitivity of 100% at a cut-off 0.014 (AUC = 0.927, ***p* = 0.005,** Fig. 1C).

**Table 2:** Association between miR-34a expression and the clinical characteristics of the AML patients

Patients’ characteristics	miR-34a expression	*p* value
Age		
<37 years	0.13 (0–66.6)	0.870
>37 years	0.09 (0–32.12)	
TLC (×10^9^/L)		
<27	0.14 (0–32.12)	0.371
>27	0.07 (0–27)	
HB (g/dl)		
<7.7	0.07 (0–66.6)	0.441
>7.7	0.13 (0–32.12)	
Platelets (×10^9^/L)		
<34.5	0.18 (0–32.12)	0.031
>34.5	0.03 (0–25.2)	
PB blast (%)		
<50%	0.092 (0–32.12)	0.864
>50%	0.11 (0–27.3)	
BM blast (%)		
<72.5%	0.16 (0–32.12)	0.117
>72.5%	0.03 (0–27.3)	
FLT-3		
Wild	0.095 (0–32.12)	0.629
Mutant	0.13 (0–27)	
NPM		
Wild	0.07 (0–32.12)	0.406
Mutant	0.02 (0–27)	
Organomegaly		
Negative	0.13 (0–32.12)	0.315
Positive	0.03 (0–66.6)	
LNs involvement		
Negative	0.12 (0–32.12)	0.252
Positive	0.03 (0–27)	
Translocations		
Negative	0.1 (0–32.12)	0.149
inv 16	0.32 (0.03–6.8)	
t (8;21)	0.02 (0–1.28)	
t (9;22)	0.16	
t(8,21), t(9,22)	5.49	
BM cellularity		
Normocellular	0.057 (0–9.6)	0.548
Hypocellular	0.23 (0.01–66.6)	
Hypercellular	0.12 (0–32.12)	
FAB		
M1	0.01 (0–0.64)	0.071
M2	0.13 (0–66.6)	
M4	0.15 (0.01–32.12)	
M5	0.16 (0–5.49)	
M7	0.012 (0.01–0.6)	
IPT		
Myelo	0.06 (0–66.6)	0.227
Mono	0.68 (0–5.5)	
Myelomono	0.23 (0–32.12)	
megakaryoblastic	0.3 (0.01–0.6)	
Risk		
Favorable	0.19 (0–9.6)	0.251
Intermediate	0.06 (0–32.12)	
Adverse	0.16 (0–27)	
Early complete remission		
Negative	0.02 (0–0.16)	0.044
Positive	0.12 (0–32.12)	
Late complete remission		
Negative	0.02 (0–0.01)	0.267
Positive	0.05 (0–0.17)	
Response to treatment		
Complete remission	0.12 (0–32.12)	0.002
Refractory	0.002 (0–0.01)	
Relapse		
Negative	0.13 (0–32.12)	0.952
Positive	0.129 (0–2.94)	
Death		
Negative	0.07 (0–9.6)	0.488
Positive	0.13 (0–32.12)	

Note: BM: bone marrow, CR: complete remission, FAB: The French-American-British, FLT-3: FMS-like tyrosine kinase 3, HM: hepatomegaly, HB: haemoglobin, HSM: hepatosplenomegaly, IPT: immunophenotyping, LN: lymphadenopathy, NPM: Nucleophosmin, PB: peripheral blood, PLT: platelets, SM: splenomegaly, TLC: total leukocyte count.

### Impact of miR-34a expression on the survival rates of the AML patients

**Figure 2:**
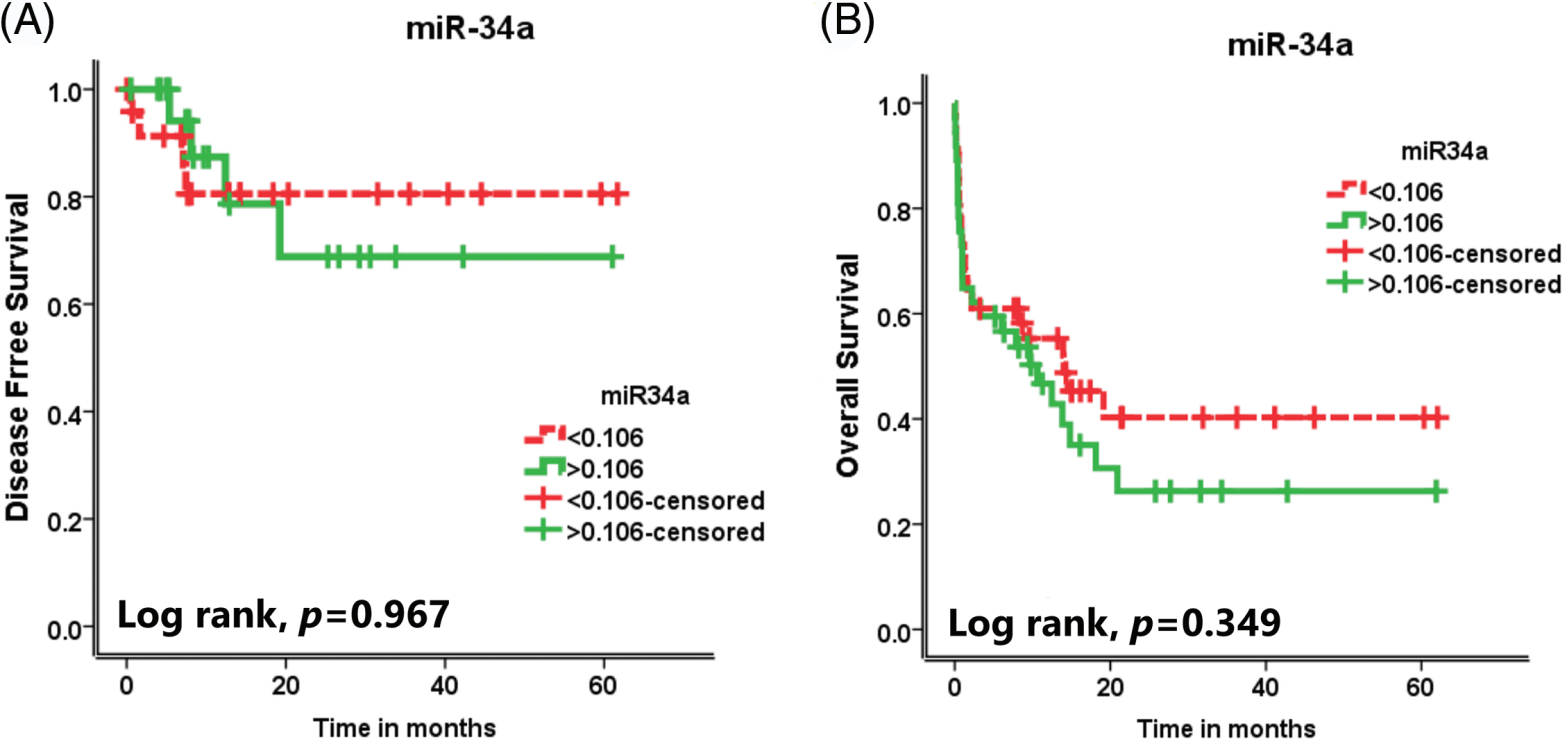
The effect of miR-34a expression on (A) the disease-free survival (DFS) rates and (B) the overall survival (OS) rates of the AML patients.

There was no significant association between miR-34a expression and the DFS rate or the OS rate of the assessed AML patients ( p =0.967 and p =0.349 , respectively, Figs. 2A, 2B).

## Discussion

It has been reported during the past few years that microRNA had an important function in the pathogenesis and development of AML [11–14,24]. Many researchers investigated the role of the miR-34 family in AML, especially miR-34a, which had been proven to be a tumor suppressor microRNA and could be used as a useful therapeutic target for AML [25–28]. However, its role in the BM of AML patients is still unclear.

The current study demonstrated that miR-34a was significantly downregulated in the BM aspirate of AML patients in the health group. In addition, The ROC curve analysis showed that miR-34a can differentiate between AML patients and control subjects with a specificity of 58.3%, sensitivity of 69.5%, and AUC of 0.707. These data are in agreement with that reported by Huang et al. [25], who found a significant downregulation of the plasma level of miR-34a in AML patients compared to the control group. Also, in line with our data, they showed that miR-34a could be used as a diagnostic biomarker for AML patients with a 73.5% sensitivity and 81.7% specificity. Similarly, Liu et al. [26] reported a significantly decreased level of miR-34a in the BM mononuclear cells of patients with AML in the control group. Moreover, it had been proposed that miR-34a was significantly downregulated *in-vitro* in leukemia cell lines [27].

To the best of our knowledge, only a few studies investigated the prognostic value of BM miR-34a in AML. The present data confirmed the fact that miR-34a expression had a significant role in evaluating response to treatment through accurately detecting ROC analysis. patients who achieved early CR (before day 14) had increased levels of miR-34a compared to those who did not achieve early CR. At the end of the treatment, patients who were refractory to therapy showed decreased levels of miR-34a compared to those who achieved CR with a specificity of 75% and a sensitivity of 100% at a cut-off of 0.014. These data are in concordance with that of Liu et al. [26], who reported that the expression level of miR-34a in the [26] BM of AML patients was higher in the complete remission group compared to the control group. In addition, Li et al. [28] proposed that increased miR-34a is expression associated significantly with a high CR rate, long event-free survival time, and OS time in elderly AML patients. Rücker et al. [29] also reported that patients with low miR-34a expression and TP53 alterations showed an increased incidence of resistance to chemotherapy and poor outcome of the treatment. In an interesting study performed by Wang et al, who reported that induced expression of miR-34a in T-cell immunoglobulin mucin-3 (TIM-3) positive leukemia stem cells (LSC), inhibits the clonogenic proliferation, tumor progression, and metastasis of leukemia. Therefore, miR-34a could be a novel therapeutic agent against LSC [30].

There were many mechanisms proposed to explain the downregulation of miR-34a in AML including that miR-34a is a downstream target of P53, TAp73, and TAp63, therefore, dysregulation of these genes could result in inhibited transcription of miR-34a [31–33]. Similarly, MYCN and STAT3 significantly increased in AML and were reported to be negative regulators for miR-34a transcription [31,34]. Moreover, the Long non-coding RNAs (lncRNAs) TUG1 was found to epigenetically inhibit miR-34a expression through increasing EZH2 (enhancer of zest homolog 2) that promotes tumor progression and aggressiveness [35]. Additionally, Wang et al reported that increased levels of lncRNAs-ANRIL in AML patients could significantly induce cancer development and progression through suppression of miR-34a expression, which in turn results in Histone deacetylase 1 (HDAC1) mediated epigenetic downregulation of ASPP2 [36]. Li et al. concluded also that miR-34a was downregulated in AML patients through increased circulating RNA (circ-POLA2) which results in increased cancer cell proliferation and metastasis [37].

Strangely, the present study showed that there was no significant association between miR-34a expression and the DFS rate or the OS rate of the assessed AML patients. These data are contradictory to that reported by some previous studies that decreased miR-34a expression associated with shorter OS and DFS of AML patients [25,28]. This discrepancy in the results could be explained by Rücker et al. [29], who reported that patients who had high miR-34a expression together with intact TP53 showed inferior overall survival (OS), whereas in patients with high miR-34a expression and TP53-biallelic-alteration showed better OS. Accordingly, all AML patients who were included in the study were not assessed for TP53 mutations which could be the reason for that discrepancy. miR-34a was demonstrated as a direct target of p53, which is recognized as a tumor suppressor gene. Therefore, TP53 aberrations were associated with decreased miR-34a expression in AML patients, denoting that loss of the p53-miR-34a signaling pathway could be a triggering factor for leukemia development [16].

Taken together, miR-34a is an important regulator of leukemogenesis through different mechanisms including inhibition of CDK4, MYB, and SIRT1 expression, and/or suppression of B-Myb and E2F1 expression, leading to cell cycle arrest in cancer cells [38,39]. Moreover, it had been reported that miR-34a induced cell apoptosis and hindered autophagy by inhibiting the expression of high-mobility group protein 1 (HMG-1) or Histone deacetylase 1 (HDAC1) enzyme [40]. Additionally, MiR-34a was reported to be a downstream target of the C/EBPα gene which has a critical function in the development and progression of AML [41,42]. Therefore, upregulating miR-34a in AML patients with C/EBPα mutations could be an effective line of treatment [42]. Furthermore, miR-34a has an immunoregulatory role by targeting PD-L1 in AML [27]. Consistent with these results, Li and his colleagues demonstrated that cell proliferation analysis revealed that Circ_POLA2 promotes AML cell multiplication by inhibiting the release of mature miR-34a [37].

Moreover, the current data showed that there was a significant downregulation of miR-34a in patients with platelet count >34.5 × 10^9^/L in comparison to those who had platelet count <34.5 × 10^9^/L.

## Conclusion

In conclusion, miR-34a was significantly downregulated in AML patients compared to the control subjects with a significant diagnostic power of 58.3% specificity and 69.5% sensitivity. Moreover, it was significantly downregulated in patients who were refractory to therapy, and it was upregulated in patients who achieved early complete remission with a specificity of 75% and sensitivity of 100%. Therefore, the current series provides evidence that miR-34a could be a useful diagnostic biomarker for AML patients. In addition, it serves as a good indicator for response to treatment in AML patients, which could identify unresponsive cases. This will give them another chance to change the treatment modality and consequently rescue them from unneeded adverse events.

## Data Availability

All required data and materials are available upon request, except for the personal data of the patients.
